# Isolation of three novel reassortant phleboviruses, Ponticelli I, II, III, and of Toscana virus from field-collected sand flies in Italy

**DOI:** 10.1186/s13071-018-2668-0

**Published:** 2018-02-06

**Authors:** Mattia Calzolari, Chiara Chiapponi, Romeo Bellini, Paolo Bonilauri, Davide Lelli, Ana Moreno, Ilaria Barbieri, Stefano Pongolini, Antonio Lavazza, Michele Dottori

**Affiliations:** 1 Istituto Zooprofilattico Sperimentale della Lombardia e dell’Emilia-Romagna “Bruno Ubertini”, Brescia, Italy; 2 Centro Agricoltura Ambiente “Giorgio Nicoli”, Crevalcore, Bologna, Italy

**Keywords:** *Phlebovirus*, Ponticelli I virus, Ponticelli II virus, Ponticelli III virus, Toscana virus, Fermo-like virus, Sand fly, *Phlebotomus perfiliewi*, *Phlebotomus perniciosus*

## Abstract

**Background:**

Different phleboviruses are important pathogens for humans; most of these viruses are transmitted by sand flies. An increasing number of new phleboviruses have been reported over the past decade, especially in Mediterranean countries, mainly via their detection in sand flies.

**Results:**

At least five different phleboviruses co-circulated in sand flies that were collected in three sites in Emilia-Romagna (Italy) in the summer of 2013. The well-known Toscana virus (TOSV) was isolated; three new, closely related phleboviruses differing in their M segments and tentatively named Ponticelli I, Ponticelli II and Ponticelli III virus, respectively, were isolated; a fifth putative phlebovirus, related to the sand fly fever Naples phlebovirus species, was also detected. The co-circulation, in a restricted area, of three viruses characterized by different M segments, likely resulted from reassortment events. According to the phylogenetic analysis of complete genome sequences, the TOSV belongs to clade A, together with other Italian isolates, while the Ponticelli viruses fall within the Salehabad phlebovirus species.

**Conclusions:**

Results highlight an unexpected diversity of phleboviruses that co-circulate in the same area, suggesting that interactions likely occur amongst them, that can present challenges for their correct identification. The co-circulation of different phleboviruses appears to be common, and the bionomics of sand fly populations seem to play a relevant role. Such a complex situation emphasizes the need for detailed investigations of the biology of these viruses to better characterize their pathogenic potential for mammals, including humans.

**Electronic supplementary material:**

The online version of this article (10.1186/s13071-018-2668-0) contains supplementary material, which is available to authorized users.

## Background

Sand flies are hematophagous dipterans with crepuscular/nocturnal activity and a resting behaviour during daytime. Their biology is largely enigmatic due to focal distribution in occupied areas linked to the availability of breeding sites and hosts and fluctuating abundance during the active season; both characteristics hamper their sampling [1, 2].

Although sand flies are fragile, small flyers that are unable to disperse over long distances, they are important to human and animal health due to their capacity to transmit a wide range of pathogens, including protozoa such as *Leishmania* parasites and different viruses belonging to the genera *Phlebovirus*, *Vesiculovirus* and *Orbivirus* [2].

Among sand fly-borne viruses, the genus *Phlebovirus* of the family *Phenuiviridae* (previously included in the family *Bunyaviridae*), is the most important group affecting human and animal health. Most of the known phleboviruses are transmitted by sand flies, while others are transmitted by mosquitoes and ticks (i.e. Rift Valley fever virus and Uukuniemi virus). Several sand fly phleboviruses are recognized as causes of disease in humans, such as sand fly fever or “three-day fever”, the self-limiting influenza-like syndrome caused by the Sicilian and Naples viruses, and summer meningitis, often caused by the Toscana virus (TOSV) [3].

Phleboviruses are characterized by a tri-segmented negative-stranded RNA genome including an L segment (encoding the RNA-dependent RNA polymerase), an M segment (encoding the two glycoproteins, Gn and Gc) and an ambisense S segment (encoding the nucleocapsid protein, N, and a smaller non-structural protein, NS) [4].

According to the International Committee on Taxonomy of Viruses [5], the genus *Phlebovirus* includes ten viral species in the Old World and the Americas. These species are defined by serological relationships in neutralization tests [6], but the discovery of several new phleboviruses brought attention to the need to introduce new criteria for phlebovirus classification [7]. Moreover, the classification of these viruses is complicated by their reassorting ability, particularly for the M segment, as already reported between related sand fly phleboviruses in South America [7] and, more rarely, in the Old World [8, 9].

Over the past decade, a wide variety of previously unreported phleboviruses were detected, particularly in the Mediterranean basin, including Sicilian-like viruses, e.g. the sand fly fever Cyprus virus [10], the sand fly fever Turkey virus [11], a phlebovirus from Algeria [12], and the Toros virus in Turkey [13]. Also, sequences of putative viruses related to this group were detected in Cyprus and Tunisia and tentatively named Girne 2 virus [14] and Utique virus [15], respectively. Different phleboviruses of the sand fly fever Naples phlebovirus species were also isolated, such as the Massilia and Granada viruses in France and Spain, respectively [8, 16], the Punique virus in Tunisia [15], the Fermo virus in Italy [17], the Arrabida virus in Portugal [9] and the Zerdali virus in Turkey [13]. Furthermore, some putative viruses were detected in France, Cyprus and Tunisia, and tentatively named Provencia [18], Girne 1 virus [14] and Saddaguia virus [19], respectively. The phleboviruses Adana [20], Alcube [21] and Medjerda Valley [22], attributable to the Salehabad phlebovirus species, were isolated and characterized in Turkey, Portugal, and Tunisia, respectively. Sequences related to putative viruses of the latter group were also detected in field-collected sand flies in Albania and Turkey and named Adria virus [23] and Edirne virus [14], respectively.

This study aimed to describe the phlebovirus diversity in sand flies sampled in 2013 in three sites within the hilly area of the Emilia-Romagna region (northern Italy) where TOSV was previously detected in 2012 [24].

## Methods

### Sampled sites

**Fig. 1:**
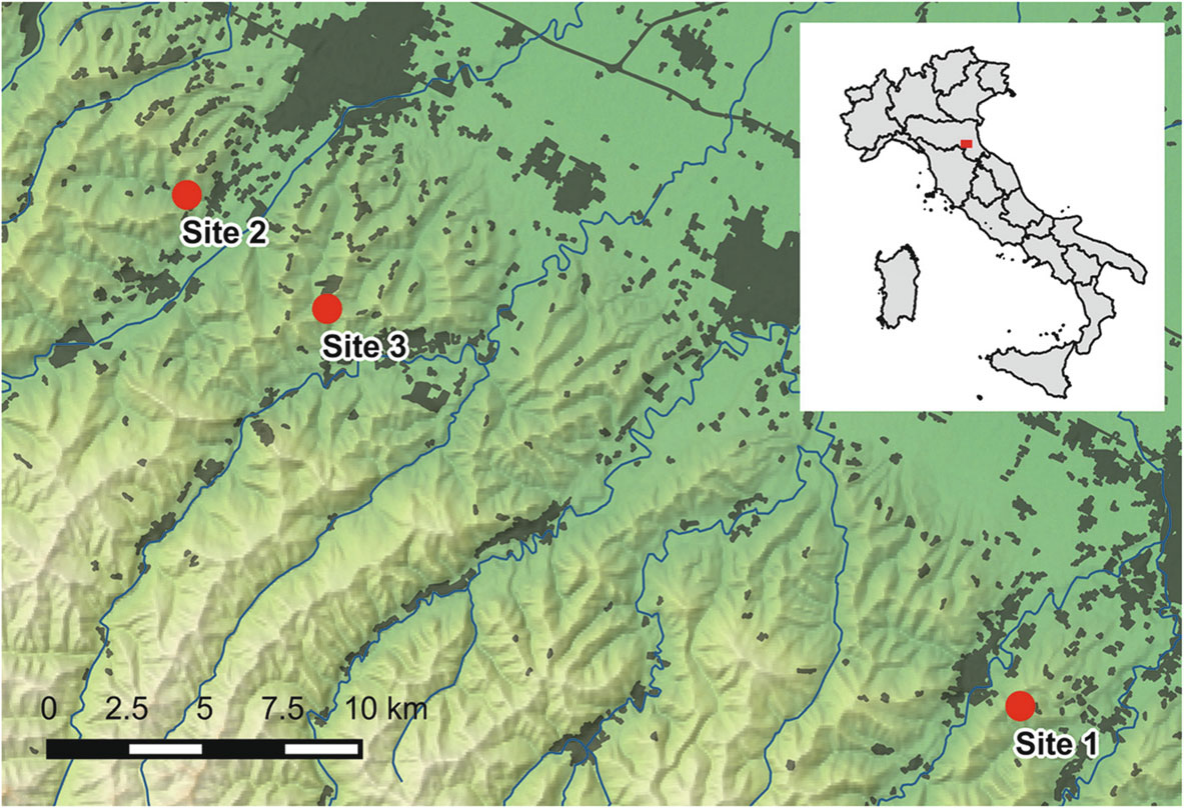
Physical map with locations of surveyed sites and position of surveyed area (red square) on a map of Italy. Urbanized areas are in gray

Three sites in which TOSV was detected in 2012 [24] were sampled in 2013. The coordinates of the sampled sites are as follows: site 1: 44°9'56"N, 11°58'9"E; site 2: 44°19'18"N, 11°38'28"E; site 3: 44°17'15"N, 11°41'46"E (Fig. 1). Site 1 was sampled twice, whereas the other sites were sampled only once. As previously described [24], sampling stations were located in the hilly area of the Emilia-Romagna region (altitude between 128 and 207 masl.). This area was characterized by cultivated fields bordered by hedges, woodlands, and badlands (Additional file 1: Figure S1).

### Sand fly sampling and identification

To collect the largest number of specimens, samples were taken in summertime, the more favourable season for sand flies in the surveyed region. Sand fly sampling was performed using carbon-dioxide-baited traps operating overnight. To maximize the number of collected sand flies, 2 to 5 traps with a minimum distance of 10 m were utilized for each site (Table 1). A subset of captured sand flies was clarified in chlorolactophenol and morphologically identified under a light microscope according to specific morphological keys [25, 26]. The remaining sand flies were stored in dry ice and used within a maximum of 5 days for virus isolation in cell culture.

**Table 1:** Sand flies that were collected and tested during the summer in 2013 concerning identified species

Site	Date	Traps	Collected	*P. perfiliewi* (M/F)	*P. perniciosus* (M/F)	Tested/Pools
Site 1	05-07-13	5	14,974	482 (40/442)	13 (0/13)	14,479/25
	23-08-13	4	5682	452 (47/405)	8 (0/8)	5222/8
Site 2	16-07-13	5	394	45 (5/40)	4 (0/4)	345/4
Site 3	23-07-13	2	2079	–	–	2079/4
Total			23,129	979 (92/887)	25 (0/25)	22,125/41

*Abbreviations*: *M* male, *F* female

### Virus isolation and purification on cell cultures

Collected sand flies were pooled by collection site; to minimize handling time (thus avoiding temperature fluctuations), and maximize the chances of isolation, large pools containing both males and females were assembled. Pools were homogenized in minimal essential medium, supplemented with penicillin and streptomycin, using a sterilized glass potter and clarified by centrifugation at 3000× *g* for 15 min. Samples were inoculated in a confluent monolayer of VERO cells (African green monkey kidney cells) at passage 170 (cell culture biobank of IZSLER, code BSCL86), incubated at 37 °C with 5% CO_2_ and observed daily for 7 days to observe the development of cytopathic effects (CPE). In the absence of CPE, the cryolysates were subcultured twice into fresh monolayers.

Isolated viruses were cultured in VERO cells under previously reported conditions and observed daily for 7 days, to characterize CPE. To exclude the presence of 2 or more viral strains in culture, selected isolates were cloned by plaque purification by 2 rounds of serial dilutions in 96-well plates (maximum dilution of 10^-8^). One of the obtained clones was re-cultured on VERO cells and subjected to sequencing.

### Virus detection and sequencing

Viral RNA was extracted from supernatant cultures that displayed CPE using TRIzol LS Reagent (Invitrogen, Carlsbad, CA, United States); cDNA synthesis was performed using random hexamers (Roche Diagnostics, Mannheim, Germany) and the SuperScriptH II reverse transcriptase (Invitrogen, Carlsbad, CA) and then tested by real-time RT-PCR for TOSV detection [27]. Samples negative for TOSV were subjected to PCR for the detection of phleboviruses (Pan-Phlebo-PCR), usibg by one reverse and two forward primers and targeting a 370-nucleotide region of the S segment [28]. The obtained amplicons were sequenced, and the sequences were utilized for virus identification by BLAST analysis against the GenBank database (https://blast.ncbi.nlm.nih.gov/Blast.cgi).

The selected viruses isolated from the cell culture supernatant were sequenced on a MiSeq Instrument (Illumina Inc., San Diego, CA, USA). To prepare DNA libraries for whole-genome sequencing, viral RNA was amplified by One-Step RT-PCR with the simultaneous amplification of all 3 viral segments. In-house-designed primers recognizing 5′ and 3′ regions of the 3 segments L, M and S, TOSV_uni (5′-GCC GGA GCT CTG CAG ATA TC, A CAC ArA GA-3′) for TOSV and PhleboV_uni (5′-GCC GGA GCT CTG CAG ATA TC, A CAC ArA G-3′) for phleboviruses, were used. To stabilize the primer in a subsequent amplification reaction by One-Step RT-PCR, a known oligonucleotide sequence was inserted in the 5′ region of the primer [29]. Briefly, 10 μl of RNA was incubated with 200 pmol primers at 65 °C for 5 min. On ice, 25 μl of 2× master mix and 1 μl of SuperScript III One-Step RT-PCR System with Platinum Taq DNA Polymerase were added to a final reaction volume of 50 μl. One-Step RT-PCR was performed with 1 cycle at 42 °C for 60 min and 1 cycle at 94 °C for 2 min. The PCR conditions were as follows: 5 cycles of denaturation at 94 °C for 30 s, annealing at 45 °C for 30 s, and extension at 68 °C for 6 min, followed by 30 cycles with the above conditions and an annealing temperature of 60 °C. PCR products were purified using NucleoSpin Gel and PCR Clean-up (Macherey-Nagel GmbH & Co, Düren, Germany). DNA libraries were prepared using a NEXTERA-XT Kit (Illumina Inc., San Diego, CA, USA) according to the manufacturer’s instructions. Pooled libraries were sequenced on a MiSeq platform (Illumina Inc., San Diego, CA, USA) using a MiSeq Reagent Kit v2 in a 250-cycle paired-end run. Data were assembled *de novo* by the NextGen DNASTAR (DNASTAR, Madison, WI, USA) application and were analyzed using Lasergene Package software (v12.0). All obtained sequences were deposited in the GenBank database under the accession numbers KU573064–KU573069 and KX388208–KX388225.

### Electron microscopy

The supernatant fluids from cell cultures showing CPEs were subjected to negative staining electron microscopy using the Airfuge method [30]. Supernatants were ultracentrifuged (Airfuge, Beckman Coulter Inc. Life Sciences, Indianapolis, Indiana, USA) for 15 min at 82,000× *g* using a rotor holding six 175-μl test tubes in which specific adapters for 3 mm carbon-coated Formvar copper grids were placed. The grids were then stained using 2% sodium phosphotungstate (pH 6.8) for 1.5 min and observed under a Tecnai G2 Spirit Biotwin transmission electron microscope (FEI, Hillsboro, Oregon, USA) at 20,500–43,000× for at least 15 min before being considered negative. Attempts to identify the observed viral particles were based on their morphological characteristics.

### Phylogenetic analysis

Obtained sequences were aligned with homologous sequences of other old-world phleboviruses transmitted by sand flies; these homologous sequences were retrieved from the GenBank database. Translated amino acid sequences were aligned using the ClustalW algorithm and alignments were refined manually; nucleotide sequences were utilized for phylogenetic analysis. The maximum likelihood approach was used to obtain a phylogenetic tree with the short-conserved sequence targets of the Pan-Phlebo PCR; the Kimura 2-parameter model with 5 discrete Gamma categories (model with the lowest Bayes factor) was selected with 1000 replicates of bootstrapping. Due to the wide diversity between complete sequences of phleboviruses, the neighbour-joining method (with distances computed using the LogDet method) was used to infer the phylogenetic tree with the M, L, and S sequences. Phylogenetic analysis, identity percentage among sequences and pairwise distance matrices were inferred using the MEGA 6 software [31]. Possible recombination events in Ponticelli sequences were checked with the Recombination Detection Program [32]. The coding sequences of the different genes (the RNA polymerase, the two glycoproteins, and the N and NS proteins) were manually concatenated, and the obtained chimeric genomes were screened by SIMPLOT software [33].

## Results

### Isolation and identification of viruses

A total of 23,129 sand flies were sampled in the 3 monitored sites, and a subsample of 1004 specimens was identified using morphological characteristics; of these, 979 were *Phlebotomus perfiliewi* and 25 were *Phlebotomus perniciosus* (Table 1). The remaining sand flies were pooled, depending on abundance, in 41 pools, and 23 of these pools showed a CPE on VERO cells (Table 2).

**Table 2:** Virological results of pools showing CPE, with reference to GenBank accession numbers of obtained sequences

Sampling	Pools	TOSV PCR	Phlebo PCR	Ponticelli virus	Fermo-like virus
Site 1/Jul	181135-1	–	+	KY354371	KY354388
	181135-2	–	+	KY354372	KY354389
	181135-3	–	+	KY354373	
	181135-4	–	+		KY354390
	181135-8	–	+	KY354374	
	181135-9	–	+	KY354375	KY354391
	181135-10	–	+	KY354376	
	181135-12	–	+		KY354392
	181135-13	–	+	KY354377	
	181135-14	+	np		
	181135-15	+	np		
	181135-16	–	+	KY354378	KY354393
	181135-17	–	+	KY354379	KY354394
	181135-22	–	+	KY354380	
Site 1/Aug	220116-1	–	+	KY354381	
	220116-2	–	+	KY354382	
	220116-3	+	np		
	220116-5	+	np		
	220116-6	–	–	KY354383	
	220116-8	–	+	KY354384	
Site 2/Jul	189826-4	–	+	KY354385	
Site 3/Jul	194246-2	–	+	KY354386	
	194246-4	–	+	KY354387	

*Abbreviation*: *np* not performed

Cell culture supernatants with CPE were subjected to biomolecular analysis, and 4 of these samples, obtained from sand flies caught in site 1 on 2 distinct days, were TOSV positive using real-time RT-PCR; these samples were then directly subjected to complete genome sequencing (Table 2).

**Fig. 2:**
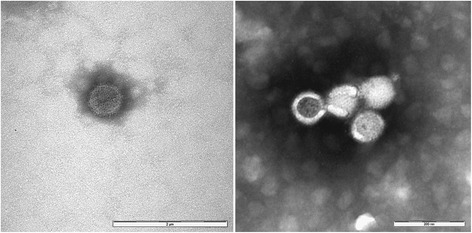
Ultramicrograph of virions observed in the supernatant of VERO cells inoculated with ground sand flies. Round particles of 80–100 nm with envelopes and tiny projections on the surface are shown. Negative staining of NaPt 2% (pH 6.8) observed with a TEM FEI Tecnai G2 Spirit Bio-twin

**Fig. 3:**
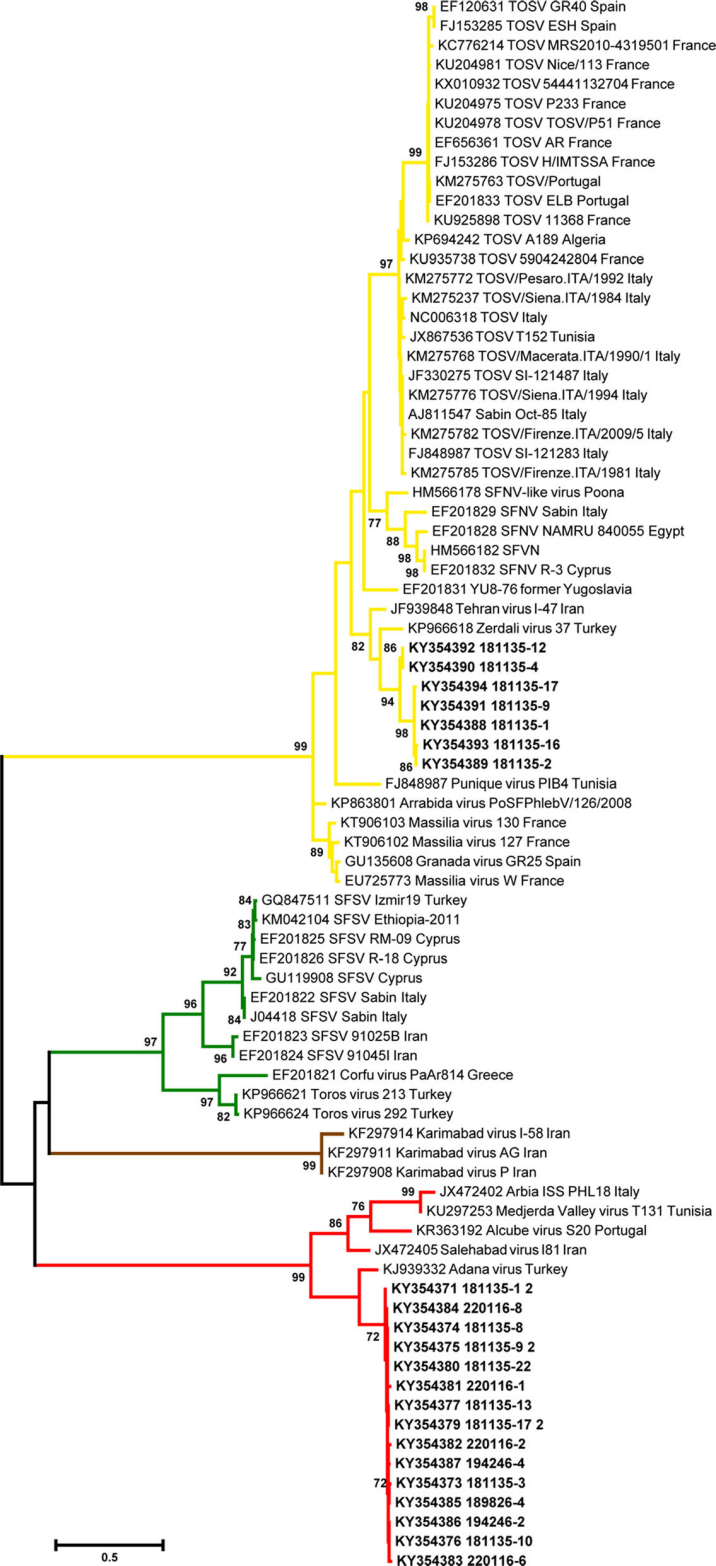
Phylogenetic tree of the field-detected S-segment sequences obtained by Pan-Phlebo PCR [28] and homologous sequences of Old-World sand fly-transmitted phleboviruses, obtained with the maximum likelihood method. Red: Salehabad phlebovirus species; green: sand fly fever Sicilian phlebovirus species; brown: Karimanbad virus; yellow, sand fly fever Naples phlebovirus species; sequences from this study in bold. Only bootstrap values over 70% are shown near the respective branches. *Abbreviations*: SFNV, sand fly fever Naples virus; SFSV, sand fly fever Sicilian virus; TOSV, Toscana virus

The presence of viruses causing CPE in TOSV-negative cell cultures were proved by electron microscopy which detected nearly spherical particles of 80–100 nm with envelopes and small projections on the surface, morphologically resembling viruses of the *Phenuiviridae* family (Fig. 2). The same cell cultures (CPE present/TOSV-negative) were tested with Pan-Phlebo PCR, and all results were positive. Sequencing of the obtained amplicons yielded 24 sequences (Table 2), grouped in 2 separate clades in the phylogenetic tree (Fig. 3). Seventeen similar sequences were attributable to a new virus, tentatively named Ponticelli virus, according to the name of a village close to one of the sampling sites in which the virus was detected (GenBank: KY354371–KY354387). The alignment of 347 positions of 15 of these sequences showed an average identity of 99.0% (ranging between 97.7–100%). These sequences clustered within a well-supported clade containing sequences of the Salehabad phlebovirus species in the phylogenetic tree (Fig. 3).

The remaining 7 sequences (alignable over 336 nucleotides, nt) were attributable to a second virus and showed an average identity of 95.1% (ranging from 100% to 89.9%) (GenBank: KY354388–KY354394). These sequences clustered within a well-supported clade including the sand fly fever Naples phlebovirus species. Interestingly, 132 nucleotides of this last group of sequences partially overlapped with 3 sequences of the Fermo virus (GenBank: LT575235–LT575237). In fact, these 2 groups of sequences showed an average identity of 97.0%, with values ranging between 96.2–97.7%.

### Full genome sequences

Deep sequencing was applied to the 4 isolates with TOSV-positive PCR results. For 2 of these isolates, a TOSV genome was obtained, while for the remaining 2, a genome attributable to the Ponticelli virus was obtained. Identity from 99.8% to 100% was obtained between sequences of the different segments of the 2 TOSV isolates: more precisely, 100% for 3413 nt of the M segment (GenBank: KU573065, KU573068), 99.8% for 5953 nt of the L segment (GenBank: KU573064, KU573067) and 99.8% for 1632 nt of the S segment (GenBank: KU573066, KU573069). In the phylogenetic tree obtained, sequences of all segments fell within the already described lineage A, along with other Italian sequences [13, 14].

Other 4 isolates from site 1 and site 3 were selected and subjected to deep sequencing, which resulted in Ponticelli virus genome identification and increased the number of Ponticelli virus sequences obtained to 6. All the obtained sequences of the L segment (GenBank: KX388211, KX388214, KX388217, KX388223, KX388220 and KX388208) and of the S segment (GenBank: KX388213, KX388216, KX388219, KX388225, KX388 and KX388210) were closely related: the average identity of 5442 nucleotides of the L segment sequences was 98.7% (ranging from 98.3% to 99.6%); the average identity of 1266 aligned nt of the S segment sequences was 99.7% (ranging from 98.0% to 99.3%). Among the M segment sequences, 4 showed high rates of identity, i.e. the alignment of 4063 nt revealed an average identity of 98.8%, ranging from 98.5% to 99.4% (GenBank: KX388212, KX388215, KX388218 and KX388224). In contrast, the other 2 M segments (GenBank: KX388209 and KX388221, 583 nt sequence) differed from each other (identity 73.9%) and the other isolates (average identity of 75.8% and 76.5%, respectively). Therefore, 3 M segments were observed and differentiated into 3 viruses tentatively named Ponticelli I, Ponticelli II and Ponticelli III virus, respectively.

**Fig. 4:**
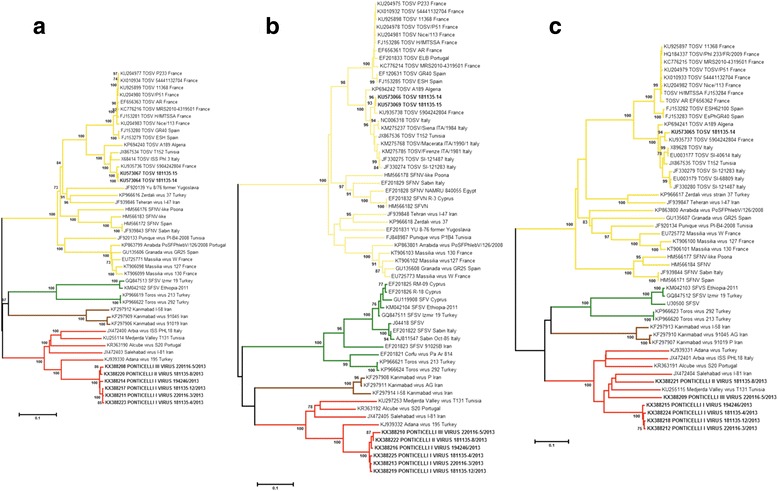
Neighbor-joining trees obtained using genome sequences from this study and homologous sequences of sand fly-borne phleboviruses from the Old World available on GenBank: **a** L segment; **b** N gene of the S segment; **c** M segment

The phylogenetic analysis of the 3 segments showed that the Ponticelli viruses clustered together with sequences of viruses of the Salehabad phlebovirus species. The L and S segment trees shared a similar topology, in which Ponticelli viruses were more similar to the Adana virus (Fig. 4a, b, Additional file 2: Figure S2a). In the M segment trees obtained using the complete alignment, the one based on the shortest M segment available or sequences encoding the 2 glycoproteins (Gn and Gc), the viruses were closer to the Salehabad and Medjerda Valley viruses (Fig. 4c, Additional file 2: Figure S2b-d). The pairwise distances obtained for the L and S segments confirmed the Adana virus as the closer virus to the Ponticelli viruses. The nucleotide sequences differed by 19.6% in the L segment, 16.5% in the N gene, and 17% in the NS gene (Table 3). These values were lower for the translated amino acid sequences, i.e. 5.1% for the L segment, 4.6% for the N gene, and 4.8% for the NS gene (Table 3). However, the 3 M segments showed variable differences within the Salehabad phlebovirus species in both the nucleotide (range 23.0–33.7%) and amino acid (range 12.4–36.4%) sequences, displaying the smallest differences compared with the Salehabad and the Medjerda Valley viruses (Table 4). The heterogeneity of M segments was evident in similarity analysis of the concatenated genes of the Salehabad phlebovirus species, which showed smallest similarity scores in portion relative to the Gn and Gc sequences (Additional file 3: Figure S3).

**Table 3:** Pairwise distance matrices of sequences of the L segment, the N gene and the NS gene of Salehabad phlebovirus species. The upper-right matrix region presents the percentage differences between amino acid alignments; the lower-left, between nucleotide alignments

	PONV	ADAV	SALV	ARBV	ALCV	MVV
L segment						
Ponticelli virus		5.1	15.7	16.6	17.4	14.5
Adana virus	19.6		14.5	15.1	15.5	14.1
Salehabad virus	26.1	25.2		10.5	11.0	9.7
Arbia virus	26.6	26.0	23.2		10.7	3.8
Alcube virus	27.1	26.5	23.9	23.3		9.8
Medjerda Valley virus^a^	24.9	25.3	22.6	19.1	23.3	
N gene						
Ponticelli virus		4.6	20.3	20.3	20.3	20.3
Adana virus	16.5		19.6	18.3	18.3	18.3
Salehabad virus	28.8	27.9		11.8	9.8	11.8
Arbia virus	27.9	28.1	24.2		7.2	0.0
Alcube virus	28.4	26.6	22.5	21.9		7.2
Medjerda Valley virus	27.7	28.6	23.8	6.9	21.2	
NS gene						
Ponticelli virus		4.8	24.9	28.2	28.2	31.1
Adana virus	17.0		25.3	28.9	29.0	31.9
Salehabad virus	30.0	32.5		30.0	30.6	28.2
Arbia virus^a^	31.0	31.9	32.3		27.1	2.4
Alcube virus	31.4	32.3	33.3	33.0		25.6
Medjerda Valley virus	31.7	31.3	33.1	32.3	29.0	

^a^Partial sequences

*Note*: GenBank accession numbers: Ponticelli virus consensus sequences; Adana virus (ADAV), KJ939330 (L), KJ939332 (N, NS); Salehabad virus (SALV), JX472403 (L), JX472405 (N, NS); Arbia virus (ARBV), JX472400 (L), JX472402 (N, NS); Alcube virus (ALCV), KR363190 (L), KR363192 (N, NS); Medjerda Valley virus (MVV), KU255114 (L), KU297253 (N, NS)

**Table 4:** Pairwise distance matrix of sequences of the M segment of Salehabad phlebovirus species. The upper-right matrix region presents the percentage differences between amino acid alignments; the lower-left, between nucleotide alignments

	PONVI	PONVII	PONVIII	SALV	MVV	ADAV	ARBV	ALCV
Ponticelli virus I		21.7	13.4	25.8	26.7	32.4	36.3	35.3
Ponticelli virus II^a^	24.1		17.0	12.4	16.0	23.7	23.7	24.7
Ponticelli virus III	23.9	26.1		26.1	28.1	31.4	36.4	35.9
Salehabad virus	24.6	23.0	26.5		28.5	33.4	38.1	36.4
Medjerda Valley virus	26.3	23.9	25.4	24.4		32.0	34.8	36.0
Adana virus	27.5	30.9	29.4	29.9	31.8		26.1	26.9
Arbia virus	31.1	31.8	33.7	32.8	30.4	29.4		21.3
Alcube virus	29.4	29.7	31.3	30.9	30.2	26.8	24.9	

^a^Partial sequence

*Note*: GenBank accession numbers: Ponticelli I virus (PONVI), KX388224; Ponticelli II virus (PONVII), KX388221; Ponticelli III virus (PONVIII), KX388209; Salehabad virus (SALV), JX472404; Medjerda Valley virus (MVV), KU255115; Adana virus (ADAV), KJ939331; Arbia virus (ARBV), JX472401; Alcube virus (ALCV), KR363191

Apparent recombination events were not detected in the M, L, and S segments (analyzed as N and NSs sequences) between Ponticelli viruses and homologous sequences of the Salehabad phlebovirus species (data not shown).

### Cell culture growth and purification

The observed CPE on VERO cells was characterized by a diffuse degeneration of the cellular monolayer, with a progressive rounding and subsequent detaching of cells, which appeared numerous and fluctuated in the culture medium. The Ponticelli II and Ponticelli III viruses produced a visible CPE from the third-day post-infection, which appeared complete, with the monolayer almost totally detached on the fifth day. The CPE produced by Ponticelli I was slower; it was visible from the fifth-day post-infection and was complete only after the seventh day.

One of the clones obtained by the purification protocol applied to the Ponticelli III virus (which was recorded in only 1 sample), was subjected to genome sequencing. The 3 segments of the clone had an almost complete identity (over 99.9%) to the previously obtained sequence, confirming the isolation of a single virus and the reliability of the results obtained.

## Discussion

The fast processing of field-collected sand flies allowed the detection of different phleboviruses: (i) TOSV was detected and isolated in one site; (ii) three closely related phleboviruses of the Salehabad phlebovirus species were detected and isolated in all three sampled sites, and tentatively named Ponticelli I, Ponticelli II and Ponticelli III virus, respectively (according to their different M segments); and (iii) another phlebovirus of the sand fly fever Naples phlebovirus species was detected but not isolated.

The co-circulation of phleboviruses of different species has often been recorded. For example, the Arbia virus and the TOSV were detected in Italy [34], the Saddaguia virus and the TOSV in Tunisia [35], the Massilia, the Alcube and the Arrabida viruses in Portugal [9, 21], and the Adana, the Zerdali and the Toros viruses in Turkey [13]. Data obtained confirm that co-circulation of different phleboviruses is a common condition, which implies a very dynamic situation with likely interactions between viruses. The close co-circulation of viruses differing only in the M segments is, however, an uncommon occurrence and highlights the importance of sequencing more than one isolated phlebovirus from any one site whenever possible. The sequence of the M segment of Ponticelli III was confirmed by cloning, and another Ponticelli II virus, with an almost identical M segment, was isolated from sand flies collected in two sites in 2016, one in Valsamoggia municipality (Bologna province), and in Lombardy, a neighbouring region of Emilia-Romagna. These findings demonstrate that three viruses differing in M segments were isolated; these viruses were likely the result of reassortment events, as also demonstrated by analysis of the concatenated genes. This mechanism, widely described in the family *Bunyavirida*e, particularly for the M segment, increases genetic variability through a genetic shift and drives the evolution of these viruses [36]. In sand fly-transmitted phleboviruses, the reassortment of the M segment was already reported in the New World in the Candiru phlebovirus species [7] and was detected in Europe between Massilia, Granada and Arrabida viruses [8, 9]. The reassortment capacity seems to play a primary role in bunyavirus evolution and in defining the viral pathogenicity of this family [36], as demonstrated by the different abilities of the three Ponticelli viruses to grow on VERO cells, which could indicate a difference in their ability to infect vertebrates. Moreover, the reassortment capacity must be taken into account to avoid misidentification based on serological tests [37].

According to the sequences obtained, the isolated TOSV falls within the lineage A [3, 24], which includes sequences isolated in Tuscany and other Italian regions, confirming the exclusive circulation of viruses from this lineage in Italy. The sequences of the detected, but not isolated, phlebovirus clustered with viruses of the sand fly fever Naples phlebovirus species, which includes viruses with a well-known pathogenic potential [3]. The portion of sequences of this virus alignable with the Fermo virus was small; however, considering the high identity rate, it is likely that the detected sequences could be attributable to the Fermo virus. Nevertheless, longer sequences are necessary to confirm the identification of the detected virus. The failure to isolate this virus was probably due to its low presence in sampled sand flies; in fact, it was detected in only one sample, together with the Ponticelli viruses, which probably displaced this virus from cell culture. Similarly, the Ponticelli viruses were also able to displace the growth of the TOSV in the two TOSV PCR-positive pools collected in August in site 1. Conversely, the TOSV was isolated in the two pools sampled earlier in the season.

The continuous discovery of new phleboviruses in the Mediterranean Basin indicates the complexity within this genus. This high diversity is probably linked to the intrinsic characteristics of these viruses. In particular, the error-prone nature of the RNA polymerase, induced the high level of nucleotide variation and the relatively low amino acid variation [3, 35], as recorded in the obtained sequences. Reassortment or recombination events [9] can also affect this diversity.

The biology of sand flies likely exerts an enhancing effect in generating this wide diversity. Sand flies do not fly far from their breeding sites, and dispersal below one kilometre is usually reported [1]. Thus, the sedentary habit and the focal distribution of these insects favour the geographical isolation of phleboviruses. Even the large population peaks characteristic of sand flies could favour the fast evolution of local viral strains, such as the sympatry of kindred species in a particular focus, like *Ph. perfiliewi* and *Ph. perniciosus*, both of the subgenus *Larroussius*, in this study. The difficulty of finding a vertebrate reservoir in the cycle of sand fly-transmitted phleboviruses may also be an indication of the primary role of sand flies in the persistence of the virus. Some phleboviruses were isolated from rodents in Africa [38], but for most of these viruses, only serological evidence of infection in vertebrates exists. In most cases, infected animals do not show any clinical evidence of infection. It seems that vertebrates act as amplifying hosts but not as reservoirs [38]. In fact, the ability of sand flies to vertically transmit phleboviruses has been widely described and testified by the presence of positive males in different studies; this characteristic further increases the diversifying capacity of sand flies on these viruses. Moreover, multiple infections in arthropods, which do not produce antibodies, could be a favourable occasion for reassortment of the genome segments [36]. All of these observations indicate that the evolution and diversity of phleboviruses are strictly linked to the bionomics of sand flies, and to the resulting intensive dynamics of transmission between these viruses and their vectors.

Data on subsamples identified at species level confirmed the overwhelming presence of *Ph. perfiliewi* among collected sand flies, as already recorded in this area [24], thereby showing that this sand fly is only the probable main vector of the TOSV in the surveyed area, but also the likely main host of the other viruses detected.

Persistence of the TOSV in a particular site was documented by the detection of the TOSV in site 1, which also showed positive results in the previous year [24]. The virus was detected in this site on two different days of sampling, although with lower infection rates than in 2012, demonstrating the ability of the TOSV to circulate within a particular area persistently.

Despite the TOSV being described as the major cause of summer meningitis in Italy, France and Spain, this virus remains a neglected pathogen. Other pathogens transmitted by sand flies, such as protozoa causing leishmaniasis, are often neglected, though their burdens are increasing worldwide. The growing health importance of these pathogens, which share the same vector, might allow the implementation of a common surveillance strategy.

## Conclusions

The discovery of new phleboviruses reported in this study raises the issue of their infectious potential for humans and animals, since these viruses have often been isolated from vertebrate cell lines (such as Vero cells) and several have been serologically detected in vertebrates. Viruses of the Salehabad phlebovirus species were not linked to disease, but antibodies against the Adana virus were serologically detected with a low prevalence in human and in domestic animal sera (particularly in goats and sheep) in Turkey [20] and human sera in Tunisia [22]. The absence of a specific serological test can impair the detection of phlebovirus infections. Nevertheless, indications of pathogenicity of some of these newly discovered viruses have been reported, such as the detection of a sequence attributable to Adria virus (of the Salehabad phlebovirus species) in a boy with febrile seizure in Greece [39]. Notably, the pathogenic capacity of phleboviruses may be recorded years after their first detection, e.g. the ability of the TOSV to cause disease was discovered approximately fifteen years after the virus was first isolated [40]. The presence of several sand fly-transmitted phleboviruses in a particular area seems to be a common situation in the Mediterranean basin and highlights the need to improve our knowledge of their biology, diagnosis and epidemiology. Therefore, future specific surveillance studies should focus on: (i) isolating the largest number of these viruses for differential diagnoses; (ii) better performing specific serological tests such as the seroneutralization test; (iii) correctly identifying phleboviruses; and (iv) assessing their pathogenic potential. At the same time, and for the same reasons, the biology of phleboviruses should be investigated in depth, and their presence in a particular area should be linked to undiagnosed diseases in humans and other mammals.

## Additional files

Additional file 1: Figure S1. Pictures of two of the sampled sites: site 1 (left) and site 2 (right). (PDF 342 kb)

Additional file 2: Figure S2. Neighbor-joining tree obtained using genome sequences (**a**, N protein of the S segment; **b**, complete M segment). (PDF 153 kb)

Additional file 3: Figure S3. Similarity plots of the concatenated sequences of the genes of the Ponticelli I, II, and III viruses, and another virus of the Salehabad phlebovirus species. (PDF 427 kb)

## Abbreviations

CPE: Cytopathic effect
masl: Meters above sea level
TOSV: Toscana virus
