# Supplementary figures and images for: Isolation of three novel reassortant phleboviruses, Ponticelli I, II, III, and of Toscana virus from field-collected sand flies in Italy
Source: Parasit Vectors. 2018 Feb 6;11:84. doi: 10.1186/s13071-018-2668-0 (PMC5802049; doi:10.1186/s13071-018-2668-0)

Figure S1. Pictures of two of the sampled sites: site 1 (left) and site 2 (right).

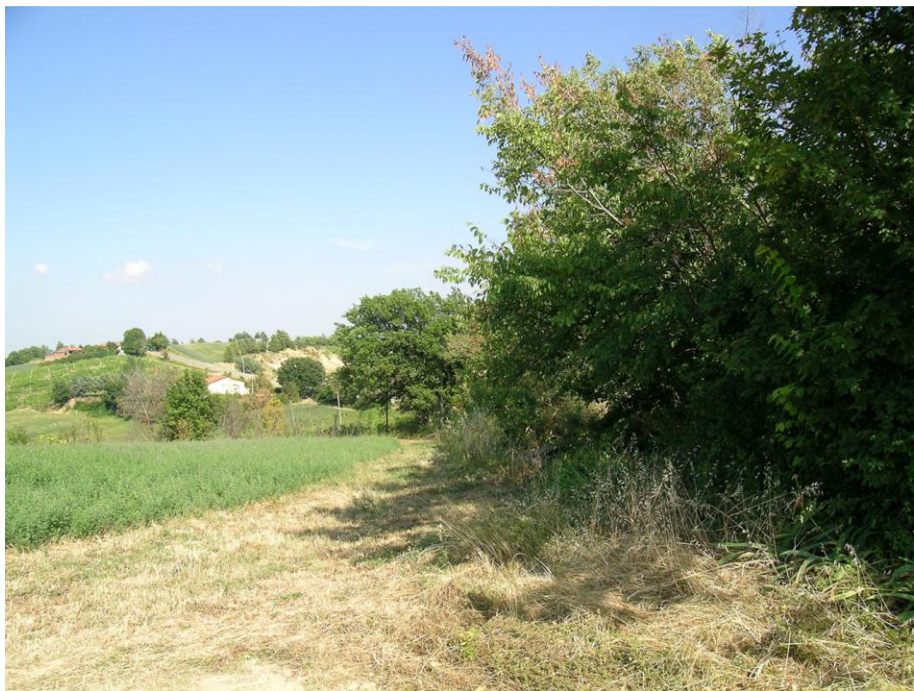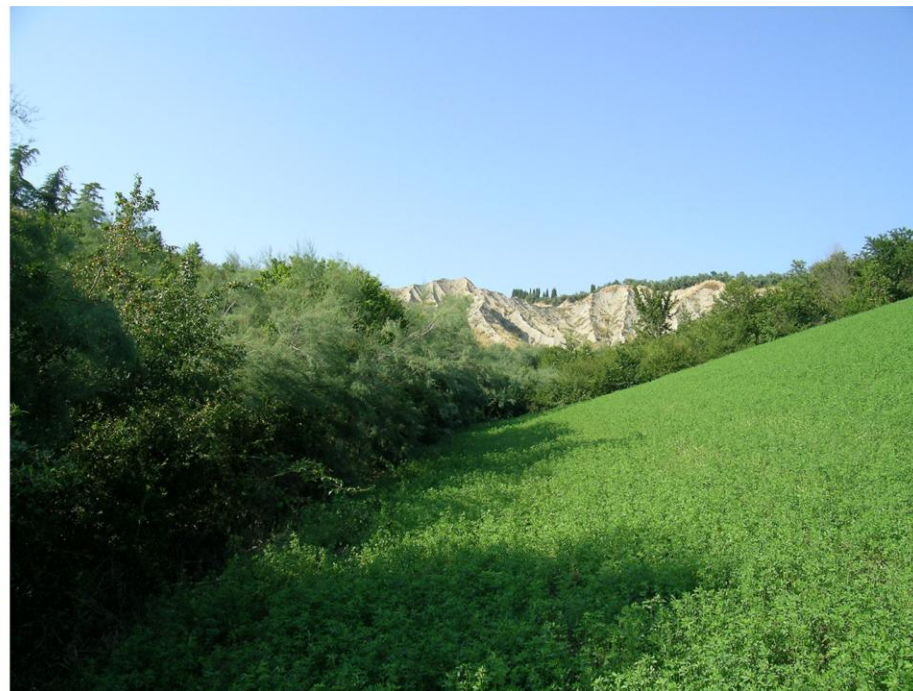

Supplement: Supplementary file 1 — Pictures of two of the sampled sites: site 1 (left) and site 2 (right). (PDF 342 kb) [file 13071_2018_2668_MOESM1_ESM.pdf]
