# Supplementary material for: Isolation of three novel reassortant phleboviruses, Ponticelli I, II, III, and of Toscana virus from field-collected sand flies in Italy
Source: Parasit Vectors. 2018 Feb 6;11:84. doi: 10.1186/s13071-018-2668-0 (PMC5802049; doi:10.1186/s13071-018-2668-0)
Supplement: Supplementary file 2 — Neighbor-joining tree obtained using genome sequences (a, N protein of the S segment; b, complete M segment). (PDF 153 kb) [file 13071_2018_2668_MOESM2_ESM.pdf]

Figure S1. Neighbor-joining tree obtained by genome sequences (a, N protein of the S segment; b, complete M segment*; c, Gn sequence of the M segment; d, Gc sequence of the M segment*).

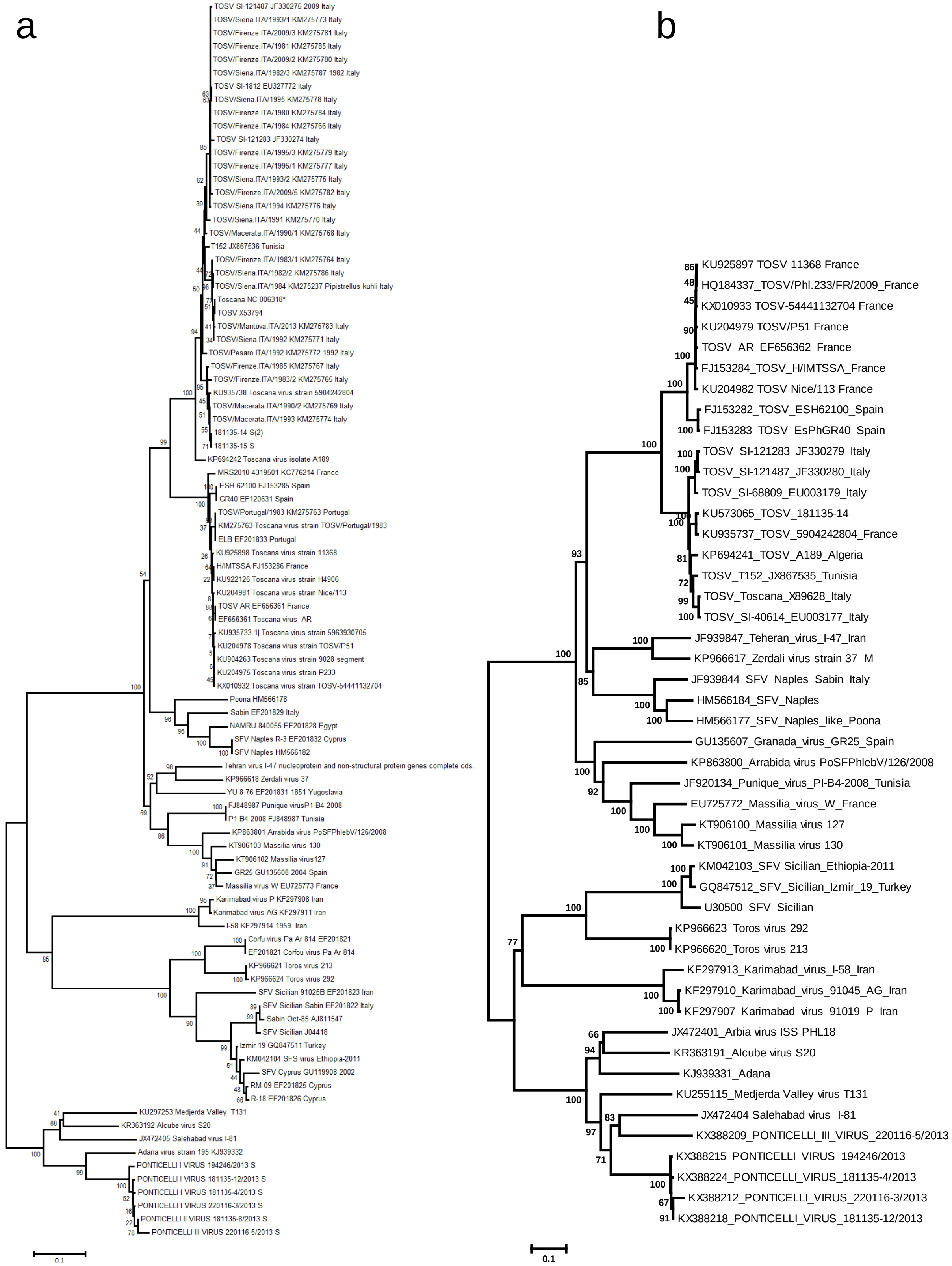

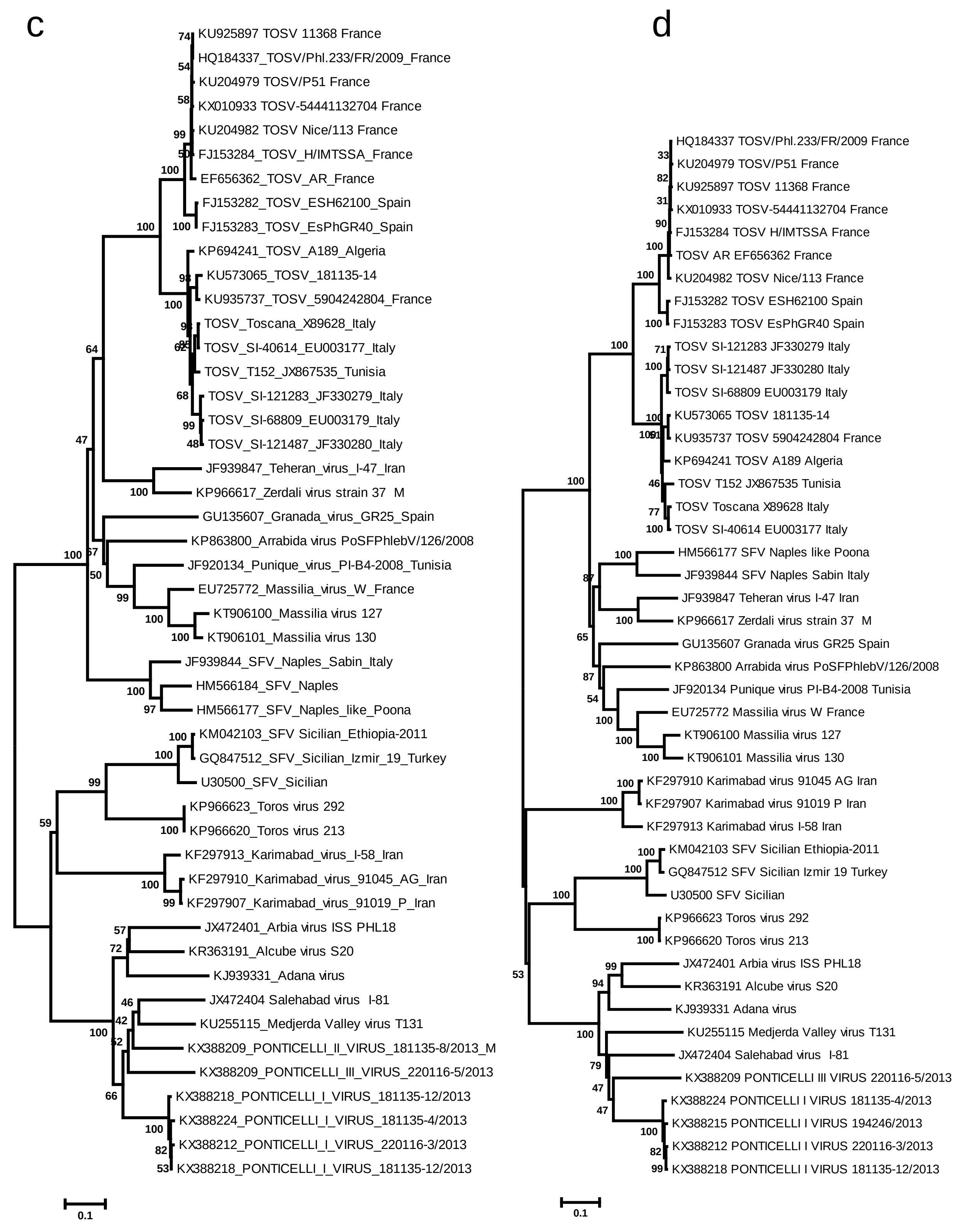

*Short sequence KX388209 not included
