# Supplementary material for: Isolation of three novel reassortant phleboviruses, Ponticelli I, II, III, and of Toscana virus from field-collected sand flies in Italy
Source: Parasit Vectors. 2018 Feb 6;11:84. doi: 10.1186/s13071-018-2668-0 (PMC5802049; doi:10.1186/s13071-018-2668-0)
Supplement: Supplementary file 3 — Similarity plots of the concatenated sequences of the genes of the Ponticelli I, II, and III viruses, and another virus of the Salehabad phlebovirus species. (PDF 427 kb) [file 13071_2018_2668_MOESM3_ESM.pdf]

Figure S3. Similarity plots of the concatenated sequences of the genes of the Ponticelli I, II, and III viruses, and other virus of the *Salehabad phlebovirus* species (window size 600 bp, step size of 10 bp, Kimura two distance model).

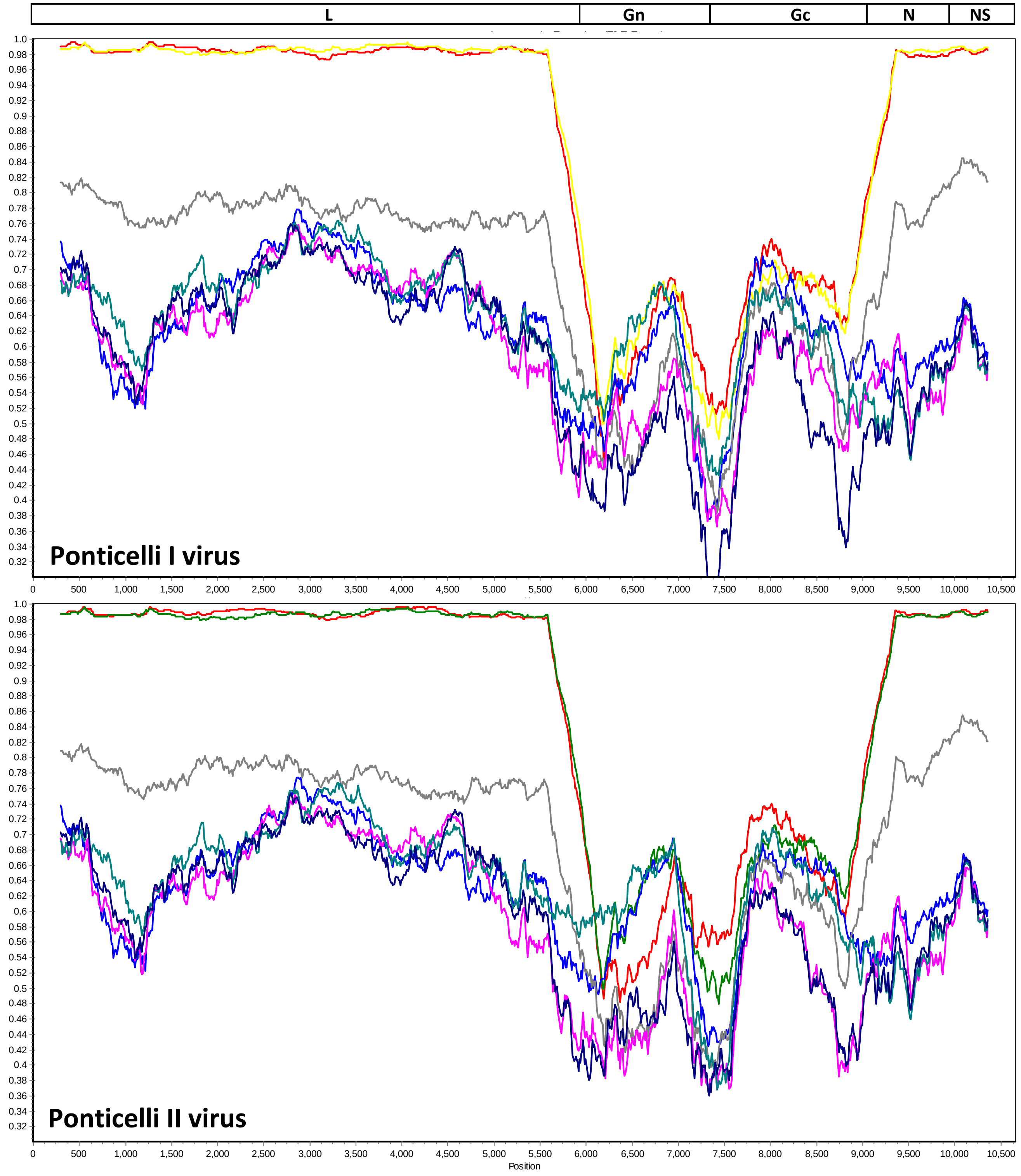

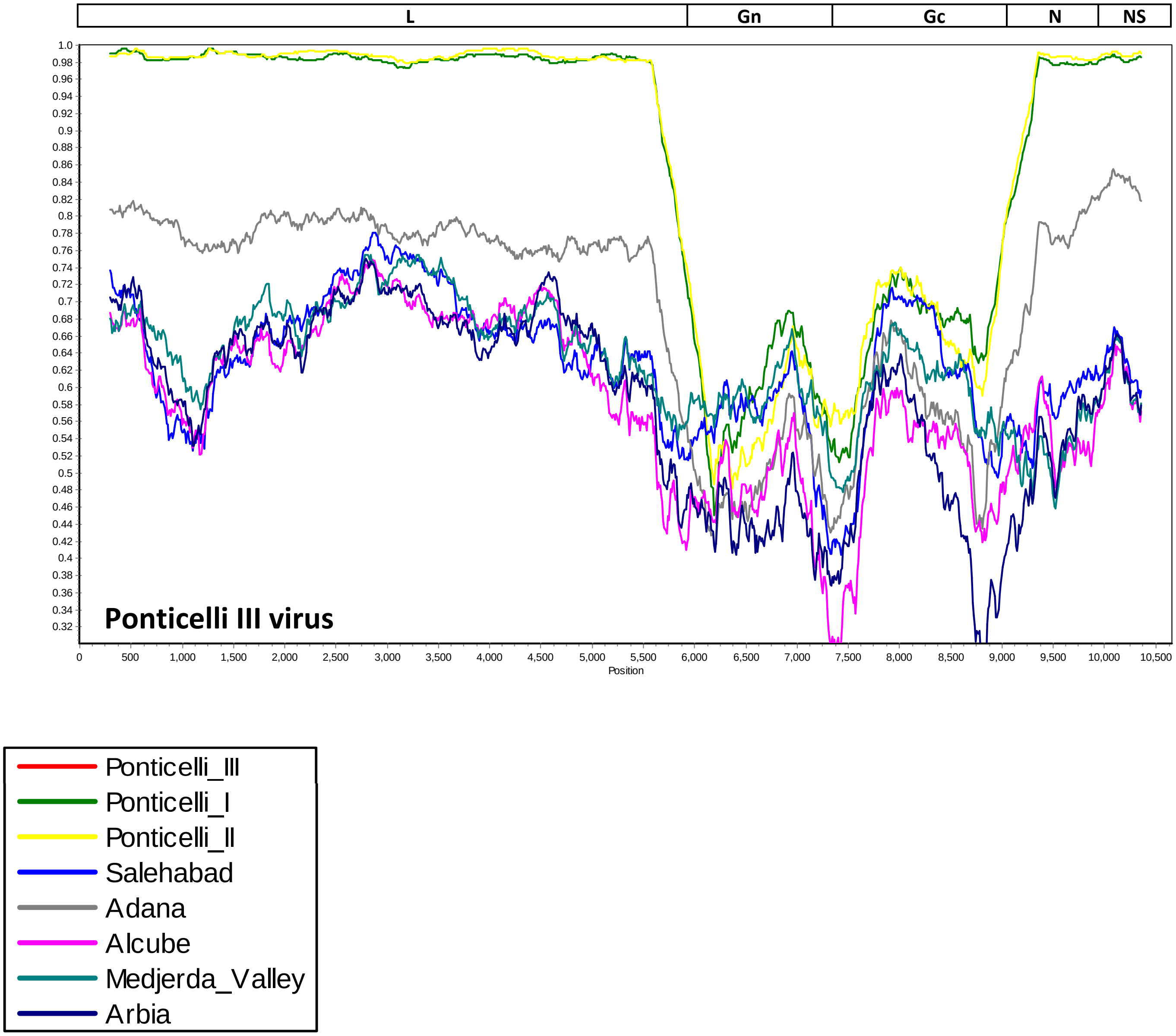

L
Gn
Gc
N
NS
Ponticelli III virus
Position
Ponticelli_III
Ponticelli_I
Ponticelli_II
Salehabad
Adana
Alcube
Medjerda_Valley
Arbia
